# Metabolic Modulation of Yogurt Fermentation Kinetics and Storage Stability by *Lactobacillus*-Starter Culture Interactions

**DOI:** 10.3390/foods14172935

**Published:** 2025-08-22

**Authors:** Meilun An, Zhi Zhao, Liang Zhao, Jianjun Yang, Haina Gao, Lele Zhang, Guoping Zhao, Baochao Hou, Jian He, Wei-Lian Hung, Baolei Li, Yangyang Yu, Shaoyang Ge, Xiaoxia Li, Ran Wang

**Affiliations:** 1 Key Laboratory of Functional Dairy, Department of Nutrition and Health, Co-Constructed by Ministry of Education and Beijing Government, China Agricultural University, Beijing 100190, China; zb20243311378@cau.edu.cn (M.A.); zxz0604@yeah.net (Z.Z.); lzhao@cau.edu.cn (L.Z.); 18811626398@163.com (J.Y.); sy20243313942@cau.edu.cn (L.Z.); 2 School of Food and Health, Beijing Technology and Business University, Beijing 100048, China; gaohaina103@126.com (H.G.); zhaogp@btbu.edu.cn (G.Z.); 3 National Center of Technology Innovation for Dairy, Hohhot 100118, China; houbaochao@yili.com (B.H.); hejian@yili.com (J.H.); hongweilian@yili.com (W.-L.H.); libaolei@yili.com (B.L.); 4 Engineering Research Center of Bio-Process, Ministry of Education, School of Food and Biological Engineering, Hefei University of Technology, Hefei 230601, China; 17855225489@163.com; 5 Beijing Heyiyuan Biotechnology Co., Ltd., Beijing 100089, China; geshaoyang@foxmail.com

**Keywords:** probiotic yogurt, fermentation kinetics, microbial viability, metabolomics

## Abstract

*Lactobacillus*-enriched yogurt is in increasingly high demand due to its health benefits, but the product stability requires an understanding of the microbial dynamics during fermentation and storage. This study investigated the interactions between probiotic pairs (*L. paracasei* L9 and *L. acidophilus* LAC) and starter culture (HYY) through fermentation kinetics, microbial viability, organic acid profiles, and metabolomics. The results demonstrated that *L. paracasei* L9 significantly increased the titratable acidity from 25.20 ± 7.01 °T to 36.56 ± 3.47 °T at 3 h and reduced the fermentation time by 0.5 h, whereas *L. acidophilus* LAC showed minimal effects. *L. paracasei* L9 achieved higher viability (8.4 lg CFU/g) via the high-affinity lactose transport and Leloir pathway, whereas the *L. acidophilus* LAC growth remained limited (6.9 lg CFU/g). The metabolomic investigation revealed the L9 + HYY upregulated glycerophospholipid metabolism and pantothenate/CoA biosynthesis to support rapid biomass accumulation. In contrast, LAC + HYY modulated the arginine and branched-chain amino acid metabolism for acid tolerance. During 21 days of storage, there were no significant differences in final TA values and lactic acid content among the probiotic supplementation groups. L9 + HYY remained stable (>9.0 lg CFU/g) by upregulating the aromatic amino acid biosynthesis and suppressing the purine/sulfur metabolism, whereas *L. acidophilus* LAC decreased to 6.02 lg CFU/g. These findings demonstrate the dual role of *L. paracasei* L9 in accelerating the fermentation and maintaining the microbial stability through metabolic reprogramming, which guides the development of improved probiotic yogurts.

## 1. Introduction

In recent years, growing consumer interest in healthy diets and functional foods has driven the increasing popularity of fermented dairy products worldwide [1]. Yogurt is one of the most representative products, and its quality largely depends on the fermentation process and composition of the starter culture [2]. Industrial yogurt production commonly relies on composite starters that consist of *Streptococcus salivarius* subsp. *thermophilus* (*S. thermophilus*) and *Lactobacillus delbrueckii* subsp. *bulgaricus* (*L. bulgaricus*), which rapidly acidify milk and develop the characteristic texture and flavor of yogurt through synergistic activity [3]. Despite their widespread use, traditional yogurt fermentation systems have several notable limitations. Because these systems primarily rely on lactic acid production via relatively simple metabolic pathways, they generate dominant flavor compounds such as lactic acid, acetaldehyde, and diacetyl, which restrict the flavor complexity and functional properties [4]. In addition, traditional starters offer limited control over the yogurt texture and often cause whey separation and a watery mouthfeel [5]. Excessive post-acidification during refrigerated storage further compromises the product stability and consumer acceptance [6]. To overcome these issues, adjunct strains with specific functional traits have been introduced into conventional systems. These strains enhance the yogurt quality by broadening the metabolic pathways, stabilizing fermentation, and improving the nutritional value by producing functional metabolites such as antioxidant peptides and vitamins [7].

Lactic acid bacteria (LAB), particularly probiotics from the genera *Lactobacillus* and *Bifidobacterium*, are widely used in functional dairy product development because they can strongly adapt to milk and have versatile metabolism. Many suitable strains for yogurt fermentation have demonstrated significant functional potential and effectively address the shortcomings of traditional starters [8]. For example, *Lacticaseibacillus paracasei* (*L. paracasei*) and *Lactiplantibacillus plantarum* can produce antioxidant peptides during milk fermentation, which enhance the functional and health-promoting properties of yogurt [9,10]. *L. casei* has been associated with the production of aromatic compounds that enhance the complexity in yogurt flavor, whereas other EPS-producing strains such as *L. paracasei* improve the texture and stability [11]. *Lactobacillus acidophilus* (*L. acidophilus*) and *Bifidobacterium animalis* subsp. *lactis* exhibit moderate acidification and stable pH profiles, so they are suitable for fermented dairy with balanced acidity and consistent flavor during cold storage [12]. These adjunct strains can be selectively combined to form multi-strain co-fermentation systems. Through metabolic complementation and synergy, they significantly improve the sensory qualities, nutritional value, and functional attributes of yogurt. Because they generally do not inhibit the growth of primary starters, they are promising candidates for next-generation, high-value yogurt products [13,14].

Although probiotic co-fermentation exhibits significant potential to enhance the yogurt quality, current research continues to predominantly focus on monitoring macroscopic fermentation parameters such as the pH dynamics, textural properties, and viable bacterial counts [14]. These conventional metrics provide valuable process control data, but they cannot elucidate the fundamental biochemical mechanisms that underlie quality improvement. There is a critical knowledge gap in our understanding of the metabolic interactions among co-cultured strains, particularly their systematic effect on non-volatile metabolite profiles and associated biochemical pathways. LAB establish complex ecological competitive/cooperative relationships that directly regulate the acidification kinetics and metabolic pathway activation [15]. Furthermore, there is no comprehensive investigation into interspecies metabolic crosstalk during refrigerated storage, so post-fermentation dynamics that govern acid-producing microbial populations and functional metabolic networks, which are crucial for product stability and functionality, remain unknown.

In this study, two representative LAB strains (*L. paracasei* L9 and *L. acidophilus* LAC) with promising application potential were co-inoculated with a commercial composite starter (HYY) to establish mixed fermentation systems. The HYY starter consists of *S. thermophilus* and *L. bulgaricus*, which rapidly acidify milk, generate characteristic yogurt flavor compounds (e.g., acetaldehyde and diacetyl), and contribute to gel formation and texture development in yogurt manufacturing [16]. We comprehensively evaluated their effects on the yogurt fermentation dynamics, bacterial viability, and non-volatile metabolite profiles during both fermentation and refrigerated storage. Using untargeted metabolomics with acidification kinetics, viable count analysis, and Kyoto Encyclopedia of Genes and Genomes (KEGG)–based pathway enrichment, we systematically investigated the strain-specific regulation of key functional modules, including amino acid, lipid, carbohydrate, and energy metabolism. This study provides new insights into the metabolic contributions and stability mechanisms of adjunct cultures throughout the fermentation–storage continuum and offers a theoretical foundation to optimize the yogurt starter formulations and develop high-value functional dairy products. This work addresses the current lack of integrated studies linking fermentation kinetics, microbial viability, and metabolomics, thereby providing novel mechanistic insights into strain-specific metabolic interactions during yogurt production and storage.

## 2. Materials and Methods

### 2.1. Strains and Cultivation Conditions

*L. paracasei* L9 (CGMCC No. 9800) and *L. acidophilus* LAC (CGMCC No. 23585) were provided by the Key Laboratory of Functional Dairy Products, China Agricultural University (Beijing, China). The commercial starter HYY, which is composed of *S. thermophilus* and *L. bulgaricus*, was supplied by the same institution. MRS broth and agar (for *Lactobacillus* spp.) were obtained from Beijing Land Bridge Technology Co., Ltd. (Beijing, China), and M17 medium (for *S. thermophilus*) was purchased from Oxoid (Basingstoke, UK).

### 2.2. Preparation of Yogurt Samples

*L. paracasei* L9 or *L. acidophilus* LAC was activated by three successive transfers in MRS broth at 37 °C, and the inoculum concentration was determined by colony enumeration, adjusted to 7.0 lg CFU/g. Activated cultures were then inoculated into sterile whole milk in combination with the commercial starter culture HYY (7.0 lg CFU/g). The control group contained only the HYY starter with no *Lactobacillus* strains. All mixtures were incubated at 42 °C until the titratable acidity (TA) reached 70 °T. After fermentation, the samples were stored at 4 °C for 21 days. Each group was prepared in triplicate, and all procedures were performed on a sterile, ultra-clean table.

### 2.3. Measurement of pH, TA, and Viable Counts

The pH of the samples was directly measured with a calibrated pH meter (Model S210, Mettler Toledo, Zurich, Switzerland). The TA was determined by titration with 0.1 M NaOH solution to the endpoint indicated by phenolphthalein and expressed as °T [17]. During fermentation, TA was measured every 2 h in the early stage and every 0.5 h when approaching the fermentation endpoint. Inflection points of TA and pH curves were identified by visual inspection of the time-course plots, corresponding to the transition from the exponential acidification phase to the plateau phase.

The viable counts of *S. thermophilus* and *L. bulgaricus* were determined by plating on M17 agar and MRS agar adjusted to pH 5.4, respectively. Then, the samples were incubated at 42 °C for 48 h. The viable counts of *L. paracasei* L9 and *L. acidophilus* LAC were determined using MRS agar adjusted to pH 6.4 after 48–74 h of incubation at 37 °C. Colony-forming units (CFU) were expressed as CFU per gram of sample [18]. All analyses were performed in triplicate using independently prepared samples.

### 2.4. Determination of the Lactic Acid Content

A 3 mL aliquot of the yogurt sample was mixed with 1.0 mL of 0.01 M sulfuric acid. The mixture was centrifuged at 12,000× *g* for 15 min at 4 °C. The resulting supernatant was filtered through a 0.22-µm aqueous membrane filter (Jinteng Experimental Equipment Co., Tianjin, China) and transferred to a high-performance liquid chromatography (HPLC) vial for analysis.

HPLC was performed using a Varian HPLC system (Varian Analytical Instruments, Walnut Creek, CA, USA) with an Aminex HPX-87H ion-exchange column (300 mm × 7.8 mm, Bio-Rad Laboratories, Hercules, CA, USA) and a refractive index detector. The column was maintained at 65 °C. The mobile phase consisted of 0.01 M H_2_SO_4_ with a flow rate of 0.8 mL/min, and the injection volume was 10 µL. The lactic acid concentration was quantified using an external standard method based on a calibration curve constructed from standard solutions [19]. Lactic acid standard solutions were prepared at concentrations of 35.93, 71.85, 143.70, 287.40, 574.80, and 1149.60 mg/L by serial dilution of a stock solution (299.2 mg/L) with 0.1% (*v*/*v*) phosphoric acid. Each concentration was analyzed in triplicate, and the repeatability of the HPLC method was assessed by calculating the relative standard deviation (RSD) of the peak areas, which was below 2% for all calibration points.

### 2.5. Extraction and Analysis of Non-Volatile Metabolites

#### 2.5.1. Sample Preparation

In total, 50 mg of freeze-dried yogurt sample was accurately weighed and extracted with 400 µL of 80% methanol that contained 0.02 mg/mL L-2-chlorophenylalanine. After pre-cooling at −10 °C, the mixture was homogenized using a high-throughput tissue grinder (Wonbio-96c, Wanbo Biotechnology, Shanghai, China) at 50 Hz for 6 min, followed by 30 min of ultrasonic extraction at 40 kHz and 5 °C. Then, the extract remained at −20 °C for 30 min to precipitate proteins and was subsequently centrifuged at 12,000× *g* for 15 min at 4 °C. The resulting supernatant was collected for further LC-MS/MS analysis [20].

#### 2.5.2. Liquid Chromatography Conditions

Chromatographic separation was performed using a UHPLC-Q Exactive HF-X system (Thermo Fisher Scientific, Waltham, MA, USA) with an HSS T3 column (100 × 2.1 mm, 1.8 µm). The column temperature was maintained at 40 °C, and the injection volume was 2 µL. The mobile phase consisted of solvent A (acetonitrile containing 0.1% formic acid) and solvent B (an aqueous solution containing 47.5% acetonitrile and 47.5% isopropanol). The gradient elution program was as follows: 0–3.5 min: B increased from 0% to 24.5%; 3.5–5.0 min: B increased to 65%; 5.0–5.5 min: B increased to 100%; 5.5–7.4 min: B remained at 100%, but the flow rate increased from 0.4 to 0.6 mL/min; and then, B decreased to 0% and re-equilibrated for 10 min with the flow rate returning to 0.4 mL/min. The samples were maintained at 4 °C throughout the chromatographic and mass spectrometric analysis [20].

#### 2.5.3. Mass Spectrometry Conditions

Mass spectrometric data were acquired using a UHPLC-Q Exactive HF-X mass spectrometer with an electrospray ionization (ESI) source that operated in both positive and negative ion modes. The ion spray voltages were set at +3500 V (positive) and −3500 V (negative) with a capillary temperature of 325 °C and a heater temperature of 425 °C. The sheath gas and auxiliary gas flow rates were 50 and 13 arbitrary units, respectively. The full MS resolution was 60,000, and the MS/MS resolution was 75,000. The mass range was 70–1050 *m*/*z*, and data were obtained in the data-dependent acquisition (DDA) mode [20].

### 2.6. Statistical Analysis

Raw LC-MS data were processed using the Progenesis QI software (v2.0, Waters Corporation, Milford, MA, USA) for baseline correction, peak detection, integration, retention time alignment, and feature extraction. The metabolites were identified based on the retention time, *m*/*z*, and MS/MS fragmentation patterns and matched against the HMDB, ChemSpider, and METLIN databases. Missing values were imputed based on the 80% rule, and total ion current normalization was applied.

Principal component analysis (PCA) and statistical analyses were performed using the MetaboAnalyst platform v5.0 (https://www.metaboanalyst.ca (accessed on 15 December 2024)) with Pareto scaling. Differential metabolites were selected based on the variable importance in projection (VIP ≥ 1.0) and *p* < 0.05 and annotated using the KEGG database. Pathway enrichment analysis was performed using the mummichog algorithm with a mass tolerance of 10 ppm. All figures were generated using GraphPad Prism 9.

Statistical comparisons were conducted using SPSS 27.0 or GraphPad Prism 8.0. First, one-way ANOVA was performed to assess the overall group differences (*p* < 0.05). When significant effects were observed, Tukey’s or Duncan’s multiple comparison tests were applied depending on the experimental design. Unless otherwise stated, all experiments were conducted in triplicate, and data are expressed as mean ± standard deviation (SD) [21].

## 3. Results and Discussion

### 3.1. Acidification Properties of Yogurt During Fermentation

**Figure 1:**
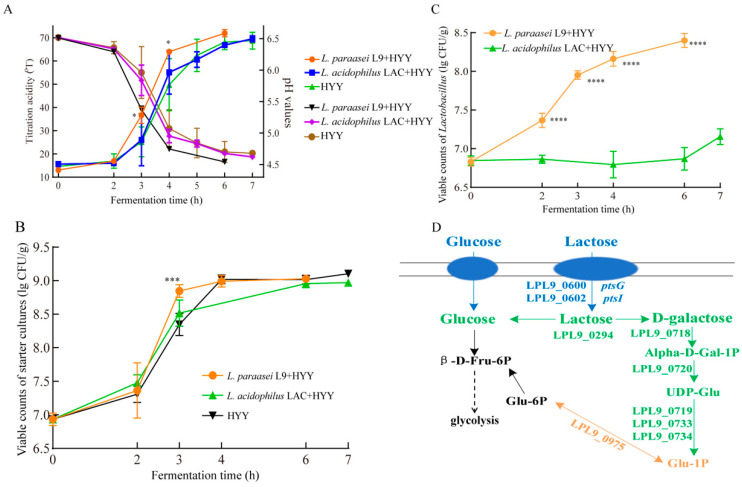
Effect of the *Lactobacillus* supplementation on acidification kinetics and microbial viability during yogurt fermentation. (**A**) Time-course monitoring of the titratable acidity development during fermentation. (**B**) Enumeration of the starter culture (HYY) viability. (**C**) Quantification of supplemented *Lactobacillus* populations. The data represent biological triplicates (mean ± SD), where statistically significant differences (*p* < 0.05) were determined using one-way ANOVA with Tukey’s multiple-comparison test. (**D**) The genomic analysis of *L. paracasei* L9 (GenBank accession: CP012148.1) revealed a comprehensive metabolic network for lactose and galactose utilization, which can be systematically categorized into four functional modules: (1) sugar uptake (blue), which involves lactose permease genes (*ptsG* and *ptsI*) and phosphotransferase system components (LPL9_0600, 0601); (2) sugar catabolism (green), which involves Leloir pathway (Galactokinase, LPL9_0718; galactose-1-phosphate uridylyltransferase, LPL9_0720; UDP-glucose 4-epimerase, LPL9_0719; and galactose-6-phosphate isomerase subunit lacAB, LPL9_0733, 0734); (3) glycolysis (black); and (4) nucleotide sugar/amino nucleotide sugar biosynthesis (orange), which involves the UDP-glucose/epimerase family genes (Phosphoglucomutase, LPL9_0975). Asterisks indicate statistically significant differences compared with the HYY control (*p* < 0.05, one-way ANOVA with Tukey’s test).

Figure 1A illustrates the acidification kinetics of yogurt fermentation systems that were co-cultured with *L. paracasei* L9 or *L. acidophilus* LAC (7.0 lg CFU/g each) and the starter culture HYY. The acidification kinetics for all treatments exhibited three distinct physiological phases, which were demarcated by inflection points in the titration curves: (1) an initial adaptation period of lag and pre-logarithmic phases (0–2 h), (2) an active acid production phase (logarithmic phase, 2–4 h), and (3) a stabilization period of late logarithmic and stationary phases (after 4 h; Figure 1A). During the logarithmic phase, acidification noticeably accelerated, which was characterized by a rapid increase in TA and a correlated steep decrease in pH values. Then, all treatment groups reached an acidification plateau (Figure 1A). The co-fermentation system supplementation with *L. paracasei* L9 exhibited distinct pro-acidification characteristics after 2 h. Specifically, at 3 h, the TA significantly increased from 25.20 ± 7.01 °T to 36.56 ± 3.47 °T (*p* < 0.05), which reduced the fermentation endpoint time (70 °T) by 0.5 h (Figure 1A). In industrial yogurt production, even a 0.5 h reduction in fermentation time can increase daily production capacity and lower energy consumption, thereby improving processing efficiency. In contrast, *L. acidophilus* LAC maintained similar TA values to the control at 3 h (27.15 ± 6.89 °T, *p* > 0.05) and showed no change in fermentation duration. This divergence in acidification profiles between the two *Lactobacillus* strains highlights their strain-specific interactions with the starter culture during milk fermentation.

The acidification kinetics of yogurt fermentation systems are fundamentally governed by the microbial acid production capacity, which is a critical technological parameter that determines the final product characteristics including the gel texture, flavor profile, and shelf stability [22]. This biochemical process exhibits clear strain specificity. The inoculation of LAB, such as *L. paracasei*, *Lactococcus lactis* subsp. *lactis* (*L. lactis*), and *Bifidobacterium* spp., during fermentation can significantly alter the fermentation timeline. These variations mainly result from differences among strains in their adaptability to dairy environments, lactose metabolism efficiency, and exponential growth kinetics. Experiments have revealed that the *L. casei* LC2W (9.0 lg CFU/g) supplementation more rapidly reduces the pH during the fermentation window of 2–4 h, shortens the time to reach the isoelectric point of casein by 30 min, and achieves 0.67% final TA [11]. Similarly, *Weissella cibaria* demonstrates synergistic effects when it is co-cultured with starter strains, where it increases the viable counts to 9.0 lg CFU/g (2.89-fold higher than axenic cultures); at 5–7% inoculation levels, *W. cibaria* significantly accelerates the early-stage acidification (0–4 h) to decrease the total fermentation time from 7 to 6 h [23,24]. In contrast, certain strains exhibit fermentation-retarding effects. For example, *B. lactis* PTCC 1631 and *B. bifidum* PTCC 1644 extend the fermentation endpoint by 10 and 15 min, respectively, compared with Yf-3331 DVS (240 min baseline). *L. lactis* UTNGt28 (1:1 with *S. thermophilus* ATCC 19258) substantially prolongs acidification, so the system reaches pH 4.5 three hours later [25]. These findings collectively establish that strains must be strategically selected to control both fermentation progression and product specifications in yogurt manufacturing, which requires careful consideration of strain-specific interactions in complex microbial consortia to optimize the culture formulation. Based on our findings, strains such as *L. paracasei* L9, which accelerate early-stage acidification and maintain high viability during storage, are more suitable for applications targeting shorter fermentation times and extended shelf stability, whereas strains like *L. acidophilus* LAC, with milder acidification and stable pH profiles, may be preferred for products requiring balanced acidity and reduced post-acidification.

### 3.2. Viable Counts of Yogurt During Fermentation

The growth kinetics of the starter cultures (*S. thermophilus* and *L. bulgaricus*) and two probiotic *Lactobacillus* strains (*L. paracasei* L9 and *L. acidophilus* LAC) were systematically monitored using selective differential media throughout the fermentation process. The inclusion of either *Lactobacillus* strain did not significantly change the final viability of the starter cultures, which maintained stable populations of 8.9–9.1 lg CFU/g (Figure 1B). This result indicates the compatibility between probiotics and traditional starters. However, the two *Lactobacillus* strains exhibited markedly different growth patterns. Their viable counts noticeably diverged after 2 h; *L. paracasei* L9 exhibited more robust proliferation and reached 7.4 lg CFU/g at this early stage. By the fermentation endpoint, *L. paracasei* L9 in the L9 + HYY co-culture achieved a final count of 8.4 lg CFU/g, i.e., a 1-log increase from the 2 h time point (Figure 1C). Meanwhile, *L. acidophilus* LAC had comparatively limited growth: it only reached 6.9 lg CFU/g in the LAC + HYY system (Figure 1C).

The ability of *Lactobacillus* to promote yogurt fermentation is closely linked to their survival rate and metabolic activity in the dairy matrix. The Leloir pathway, which converts galactose into glycolytic intermediates for ATP generation, may explain the faster acidification of *L. paracasei* L9 compared with *L. acidophilus* LAC. *L. paracasei* L9 exhibits efficient lactose utilization due to its high-affinity lactose transport system and complete Leloir pathway (Figure 1D), which enables rapid proliferation through enhanced glycolysis. This metabolic advantage accelerates the acidification and consequently shortens the fermentation endpoint by 0.5 h compared with the controls. In contrast, *L. acidophilus* LAC prefers glucose metabolism to lactose metabolism, which is attributed to its inducible β-galactosidase system and inefficient Leloir pathway [26,27]. Studies have confirmed that *L. acidophilus* metabolizes glucose at a significantly higher efficiency than lactose, where the lactic acid production in lactose media only constitutes 20–50% of that in glucose-based systems [28]. This metabolic constraint delays the growth initiation and reduces viability in milk fermentation. Furthermore, *L. acidophilus* exhibits marked pH sensitivity during early fermentation stages. *L. acidophilus* LA-5 experiences severe growth inhibition at pH 4.4, where the viable counts decreased to 9.09 lg CFU/g (62.96% of optimal conditions) [29]. This acid sensitivity and competitive disadvantages against starter cultures (e.g., *S. thermophilus* and *L. bulgaricus*) in substrate utilization can collectively limit the proliferation and acidification capacity of *L. acidophilus* LA-5 in co-culture systems.

### 3.3. Metabolite Changes During Fermentation

**Figure 2:**
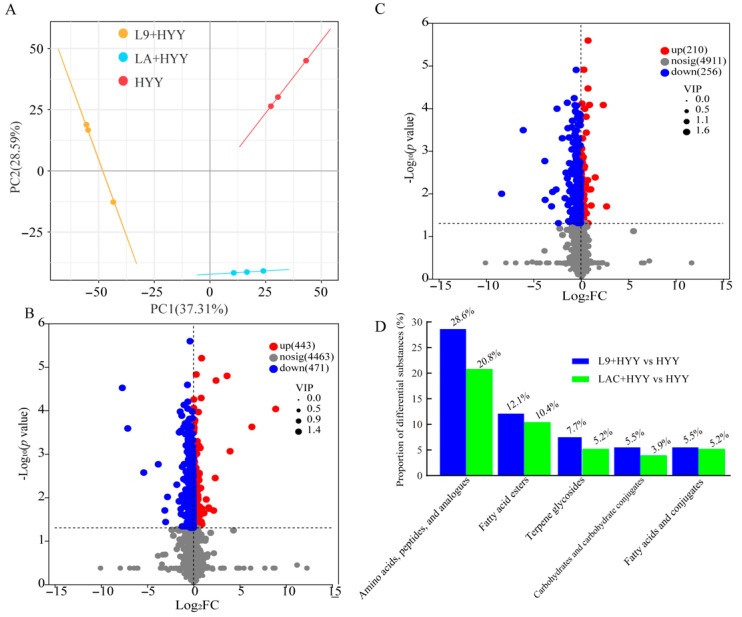
Non-volatile metabolite signatures in *Lactobacillus* co-fermented yogurt systems at the terminal fermentation phase. The comprehensive metabolomic characterization revealed distinct metabolic patterns across fermentation groups: (**A**) PCA clearly separated the metabolic profiles among experimental groups. Differential metabolite analysis identified significant alterations in (**B**) L9 + HYY and (**C**) LAC + HYY co-cultures relative to HYY control, visualized through volcano plots (log_2_ fold-change vs. −log_10_ *p*-value). (**D**) Hierarchical classification of differentially abundant metabolites according to the HMDB chemical taxonomy. Metabolites were profiled using UHPLC-Q Exactive HF-X with dual-polarity ionization (ESI^+^ and ESI^−^). Raw data were normalized to internal standards and Pareto-scaled prior to statistical processing. Significant metabolites were defined based on the following criteria: (1) VIP ≥ 1.0 from OPLS-DA models, (2) *p* < 0.05 (two-tailed *t*-test), and (3) log_2_FC > 1 or <0.5. All results represent triplicate biological experiments that were analyzed under uniform scaling conditions.

To further investigate the metabolic changes induced by adding two *Lactobacillus* strains, an untargeted metabolomics analysis was performed on yogurt samples. At the end of fermentation, 4991 metabolites were identified and quantified with high confidence based on accurate mass measurement, MS/MS fragmentation matching, and database searches (HMDB, METLIN, ChemSpider). These metabolites were classified into several major categories: 1442 lipids and lipid-like molecules; 1005 organic acids and derivatives; 565 organoheterocyclic compounds; 429 organic oxygen compounds; 313 benzenoids; 237 phenylpropanoids and polyketides; 121 nucleosides, nucleotides, and analogues; and 70 alkaloids and derivatives (Figure S1). A PCA was conducted to evaluate the overall metabolic variation among the yogurt samples. The first two principal components (PC1 and PC2) explained 37.31% and 28.59% of the total variance, respectively, which indicates that a large proportion of the metabolic differences were captured by these components (Figure 2A). The three biological replicates in each group were tightly clustered, which reflects high experimental reproducibility and data consistency. The clear separation without overlap among the three groups suggests low intra-group variance and high inter-group specificity, which reinforces the reliability of the PCA model and distinct biochemical signatures of each bacterial strain [30]. Notably, the L9 + HYY and LAC + HYY groups clearly separated from the HYY control group along both PC1 and PC2, which suggests that the addition of *L. paracasei* L9 and *L. acidophilus* LAC significantly changed the metabolic landscape of the yogurt. Different adjunct strains are likely to induce distinct shifts in metabolic pathways, and such profiling can guide the selection of strains with desired functional traits, such as targeted flavor enhancement, improved texture, or increased nutritional value.

To elucidate the strain-specific metabolic effects during milk fermentation, we conducted comprehensive metabolomic profiling to compare *L. paracasei* L9 and *L. acidophilus* LAC co-cultures with the HYY starter using stringent statistical criteria (VIP ≥ 1.0 and *p* < 0.05). The analysis revealed distinct metabolic patterns between the strains: *L. paracasei* L9 generated 914 differentially expressed metabolites (DEMs) relative to the HYY, which comprised 443 upregulated and 471 downregulated species (Figure 2B). The LAC + HYY yogurt produced 466 DEMs (210 upregulated and 256 downregulated), i.e., a 51% decrease in metabolic effect relative to the L9 + HYY yogurt (Figure 2C). The altered metabolites predominantly comprised nitrogenous compounds including amino acids, peptides and analogues (20.8–28.6% of total DEMs), lipid derivatives such as fatty acids esters/conjugates (15.6–17.6%), carbohydrates/conjugates (3.9–5.5%), and specialized metabolites including terpene glycosides (5.2–7.7%; Figure 2D). These differences in metabolite profiles gently highlight the superior capacity of *L. paracasei* L9 to diversify the milk metabolite composition, which is consistent with previous findings that certain *L. paracasei* strains can broaden the range of bioactive peptides, organic acids, and aromatic compounds in fermented dairy products [16].

### 3.4. Metabolic Pathway Analysis During the Fermentation Period

**Figure 3:**
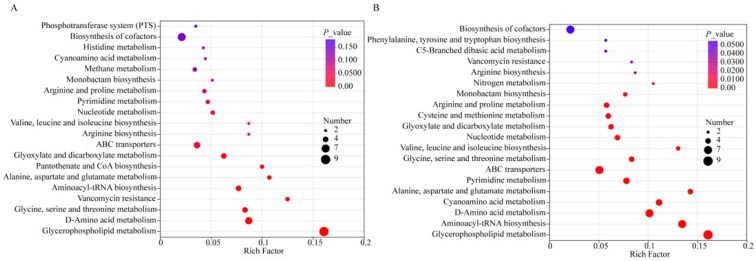
Enriched metabolic pathways in *Lactobacillus*-fermented yogurt at the terminal fermentation stage. Based on differential metabolic pathway analyses, *Lactobacillus*-supplemented yogurt (L9 + HYY and LAC + HYY) was compared with conventional HYY yogurt. (**A**) L9 + HYY and (**B**) LAC + HYY exhibited distinct pathway enrichment patterns (*p* < 0.05) in both positive and negative ionization modes. Analyses were performed in MetaboAnalyst 5.0 (KEGG database) with significance thresholds at VIP ≥ 1.0 and *p* < 0.05. The pathway importance is represented by topological impact scores (*x*-axis); the statistical significance (−log_10_ *p*-value, *y*-axis) is visualized using the bubble size (impact value) and color gradient (*p*-value magnitude).

To determine the metabolic characteristics and pathways induced by lactobacilli at the end of fermentation compared with the starter culture, pathway analysis was performed using the hypergeometric distribution algorithm in MetaboAnalyst. The relative importance of pathways in the entire network was more comprehensively characterized through a statistical analysis of Betweenness Centrality. Metabolomic pathway analysis revealed distinct adaptive strategies governing *L. paracasei* L9 and *L. acidophilus* LAC in co-fermented yogurt systems. Glycerophospholipid metabolism, D-amino acid metabolism, pantothenate/CoA biosynthesis, and glyoxylate/dicarboxylate metabolism were significantly enriched in the L9 + HYY yogurt (Figure 3A). In contrast, the following pathways were significantly altered in the LAC + HYY yogurt: biosynthesis of tyrosine and tryptophan, arginine biosynthesis, arginine and proline metabolism, and biosynthesis of valine, leucine, and isoleucine (Figure 3B).

The upregulation of pantothenate and CoA biosynthesis amplifies the acetyl-CoA flux through the tricarboxylic acid (TCA) cycle to meet increased ATP demands for rapid cell division and supports the vigorous metabolic activity [31]. In addition, upregulation of pantothenate and CoA biosynthesis can activate the glycerophospholipid metabolism, which directly facilitates a membrane biogenesis by providing essential phospholipid components (PC (16:0/18:1), GPEtn ((16:0/18:1)/(14:0/22:2)/(16:1/18:1)), and PE (18:1(11Z)/18:2(9Z,12Z)) for cellular expansion during logarithmic growth. Concurrently, the activation of glyoxylate/dicarboxylate metabolism enables efficient acetate assimilation by bypassing decarboxylation steps in the TCA cycle; this activation step is a critical feature for heterofermentative metabolism [32,33,34]. Furthermore, this metabolic rewiring optimizes the carbon conservation during energy-intensive proliferation phases. D-amino acid metabolism potentially supports remodeling decarboxylation for structural integrity during rapid biomass accumulation [35].

In contrast, LAC + HYY demonstrates evolutionary specialization for acidic niche adaptation. The enhanced amino acid biosynthesis pathways, particularly for aromatic amino acids (tyrosine/tryptophan) and branched-chain amino acids (valine/leucine/isoleucine), can improve the production of hydrophobic amino acids by altering protein–lipid interactions, thereby maintaining membrane fluidity under acidic conditions [36,37]. In addition, these amino acids can act as compatible solutes to maintain cellular osmotic balance and serve as alternative energy sources under acidic stress. The activation of the arginine deiminase (ADI) pathway generates ATP and ammonia, the latter neutralizing cytoplasmic acidity, while the accumulated proline acts as a compatible solute to stabilize macromolecules against acid-induced denaturation [38,39]. This multi-layered adaptation strategy allows *L. acidophilus* to sustain enzymatic activity and membrane function in low-pH yogurt matrices, which enhances the acid tolerance.

### 3.5. Storage Period Performance of Different Strains of Yogurt

**Figure 4:**
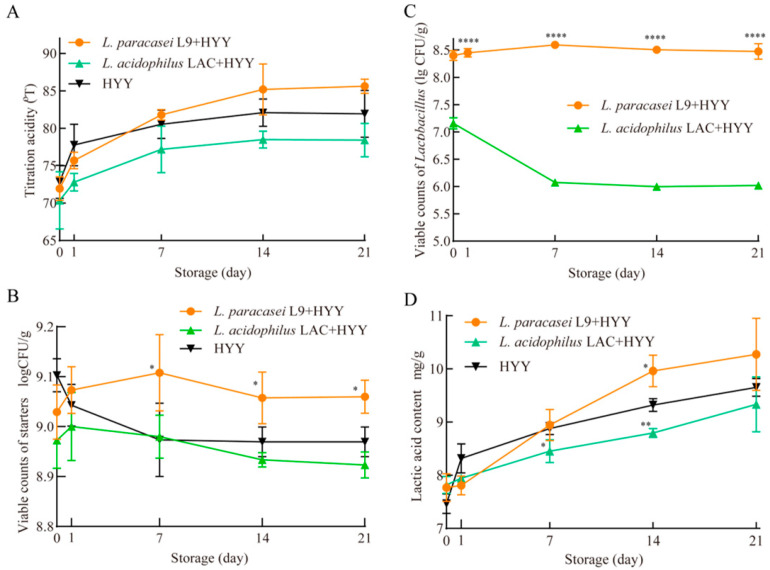
Microbial dynamics and acidification profile under refrigerated conditions. (**A**) Temporal evolution of acidification parameters throughout cold storage. Population dynamics expressed as log_10_-transformed colony-forming units: (**B**) starter culture viability and (**C**) persistence of supplemented *Lactobacillus* strains (*L. paracasei* and *L. acidophilus* LAC) during 21-day storage. (**D**) Quantitative analysis of lactic acid accumulation. The results are presented as mean values ± standard deviation from three independent biological replicates (n = 3). Microbial enumeration was performed via serial dilution plating on strain-specific culture media, where colony counts were recorded after appropriate incubation periods. Asterisks indicate statistically significant differences compared with the HYY control (*p* < 0.05, one-way ANOVA with Tukey’s test).

Figure 4 shows the evolution of TA, viability of starter cultures, and supplemented *Lactobacillus* populations in yogurt samples during 21 days of refrigerated storage (4 °C). All yogurt formulations exhibited progressive acidification during storage; the most significant TA increase occurred in the first 7 days (Figure 4A). After 21 days, the final TA values showed no statistically significant differences between probiotic-supplemented groups (L9 + HYY: 85.62 ± 0.95 °T; LAC + H YY: 78.42 ± 2.22 °T) and HYY yogurt (81.93 ± 3.12 °T) (*p* > 0.05), which demonstrates the minimal effect of the *Lactobacillus* supplementation on post-acidification. The microbiological analysis revealed distinct strain-specific preservation patterns. The L9 + HYY system demonstrated superior maintenance of starter culture viability: the counts increased during 0–7 days before stabilizing above 9.0 lg CFU/g throughout the remaining storage period and were consistently 0.1 lg CFU/g higher than the control values (*p* < 0.05; Figure 4B). In contrast, the starter viability in the LAC + HYY yogurt (8.9 lg CFU/g) matched the HYY yogurt levels. Regarding probiotic stability, *L. paracasei* L9 maintained robust populations (8.47 lg CFU/g), whereas the *L. acidophilus* LAC viability markedly decreased to 6.02 lg CFU/g (Figure 4C). As shown in Figure 4D, lactic acid content increased markedly during the first 7 days of storage and then plateaued, closely mirroring the TA trends in Figure 4A. This correlation suggests that post-acidification during refrigerated storage is primarily driven by continued lactic acid production from residual lactose metabolism.

These observations are consistent with the existing literature on the cold tolerance of *Lactobacillus*; in particular, *L. casei* strains such as PRA205 can sustain populations > 8.0 lg CFU/g after 28 days at 4 °C, i.e., they outperform less tolerant species such as *L. rhamnosus* PRA331 (7.29 lg CFU/g) [40]. The superior cold adaptation of *L. paracasei* appears to be multifactorial: (1) membrane lipid composition modifications that maintain fluidity under low-temperature stress; (2) metabolic plasticity to enable the continued production of protective metabolites (e.g., amino acids and short-chain fatty acids) and create a mutually supportive microenvironment; and (3) enhanced acid resistance mechanisms [41]. Conversely, *L. acidophilus* was particularly sensitive to the combined acid and cold stress: its viability precipitously decreased below pH 4.5, which likely explains its poorer performance in refrigerated dairy matrices [42]. These findings provide practical insights for dairy manufacturers by highlighting the importance of selecting adjunct strains that not only improve fermentation performance but also enhance product stability during cold storage. By optimizing starter culture formulations and monitoring key metabolic markers, dairy companies can extend shelf life, improve sensory quality, and deliver functional yogurt products that meet consumer expectations.

### 3.6. Metabolite Changes During Storage

**Figure 5:**
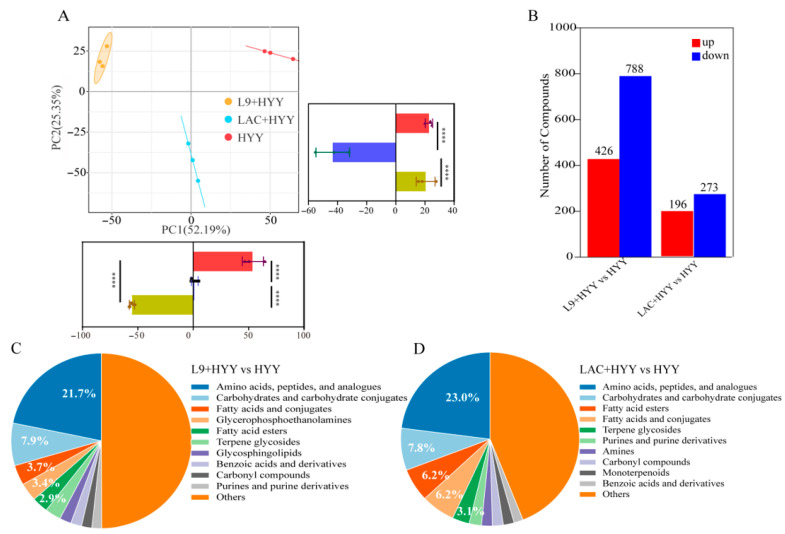
Metabolomic profiling of *Lactobacillus*-supplemented yogurt samples in 21-day refrigerated storage. (**A**) PCA delineating the treatment-specific metabolic clustering patterns. (**B**) Bar chart visualization of differential metabolites (VIP ≥ 1.0, *p* < 0.05, and |log_2_FC| > 1) after 21 days of storage at 4 °C. HMDB-based chemical categorization of storage-induced metabolic alterations in (**C**) L9 + HYY and (**D**) LAC + HYY yogurt. Analytical workflow comprised a UHPLC-Q Exactive HF-X analysis with complementary ionization (ESI ^±^); data processing included the following steps: (1) internal standard normalization, (2) unit variance scaling, and (3) multivariate modeling (OPLS-DA/PLS-DA). Metabolite significance was established through a tripartite threshold system that incorporated the following: (i) supervised modeling importance (VIP ≥ 1.0), (ii) statistical rigor (*p* < 0.05, two-tailed *t*-test), and (iii) magnitude of change (two-fold minimum difference). Triplicate biological datasets underwent identical preprocessing protocols to ensure analytical consistency. Statistical significance is indicated as follows: **** *p* < 0.0001.

To assess the strain-specific effects of *Lactobacillus* on yogurt metabolism during refrigerated storage, we conducted PCA of non-volatile metabolites from samples stored at 4 °C for 21 days. The PCA score plot revealed clear separation among the three treatment groups; the first two principal components (PC1: 52.19%; PC2: 25.35%) collectively accounted for 77.54% of the total variance and effectively captured major metabolic differences (Figure 5A). Distinct group clustering with tight intra-group aggregation and clear inter-group separation demonstrated both strong treatment effects and high experimental reproducibility. A statistical analysis of the component scores showed that the L9 + HYY group had significantly lower PC1 values (*p* < 0.0001), where LAC + HYY exhibited the lowest PC2 scores (*p* < 0.0001 vs. other groups; Figure 5A), which validated the observed group separation. Notably, metabolic patterns remained consistent with fermentation endpoint profiles and indicated sustained strain-specific effects throughout storage [43].

To further compare non-volatile metabolite changes among the groups, a bar chart of differential metabolites was constructed (Figure 5B). Metabolites were filtered based on the criteria VIP ≥ 1.0, *p* < 0.05, and |log_2_FC| > 1. In total, 1214 differential metabolites (426 upregulated and 788 downregulated) were identified in the L9 + HYY vs. HYY comparison. In contrast, only 469 differential metabolites (196 upregulated and 273 downregulated) were detected in the LAC + HYY vs. HYY comparison. HMDB classification showed both strains similarly affected the core metabolic categories (amino acids/peptides/analogues: 21.7–23.0%; carbohydrates: 7.8–7.9%), but distinct lipid modulation patterns appeared: fatty acids/esters constituted 9.9% of altered metabolites in LAC + HYY vs. 6.6% in L9 + HYY (Figure 5C,D). Strain-specific signatures included glycerophosphoethanolamine enrichment (3.4%) in L9 + HYY (Figure 5C) and terpene glycoside accumulation (3.1%) in LAC + HYY (Figure 5D), which collectively demonstrates that *L. paracasei* L9 induces more extensive and structurally diverse metabolic remodeling during early cold storage than *L. acidophilus* LAC [13].

### 3.7. Metabolic Pathway Analysis During the Storage Period

**Figure 6:**
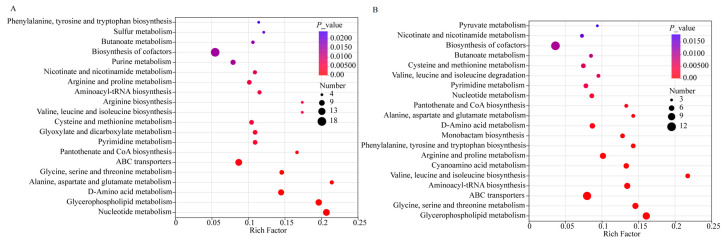
Enriched metabolic pathways in *Lactobacillus*-fermented yogurt after 21 days of refrigerated storage. A differential metabolic pathway analysis was conducted to compare *Lactobacillus*-supplemented yogurt (L9 + HYY and LAC + HYY) with conventional HYY yogurt. (**A**) L9 + HYY and (**B**) LAC + HYY exhibited distinct pathway enrichment patterns (*p* < 0.05) in both positive and negative ionization modes. Analyses were executed in MetaboAnalyst 5.0 (KEGG database) with significance thresholds set at VIP ≥ 1.0 and *p* < 0.05. The pathway importance is represented by topological impact scores (*x*-axis), where the statistical significance (−log_10_ *p*-value, *y*-axis) is visualized using the bubble size (impact value) and color gradient (*p*-value magnitude).

To elucidate the metabolic mechanisms driving *Lactobacillus*-mediated changes in microbial populations during 21 days of refrigerated storage, we performed metabolic pathway topology analysis and intermediate centrality index analysis. Significant metabolic pathways (VIP ≥ 1.0 and *p* < 0.05) were identified across different yogurt formulations. There were distinct metabolic pathway changes across different yogurt formulations. In the L9 + HYY yogurt, the following pathways were significantly upregulated: pantothenate and CoA biosynthesis and phenylalanine, tyrosine, and tryptophan biosynthesis (Figure 6A). Conversely, multiple catabolic pathways showed significant inhibition: nucleotide metabolism, glycerophospholipid metabolism, arginine biosynthesis, purine metabolism, sulfur metabolism, and butyrate metabolism (Figure 6A). For the LAC + HYY yogurt, the affected pathways consisted of glycerophospholipid metabolism; glycine, serine, and threonine metabolism; pyruvate metabolism; butyrate metabolism; and cyanoamino acid metabolism (Figure 6B).

The upregulation of pantothenic acid and CoA biosynthesis is particularly noteworthy because these compounds play pivotal roles in cellular energy metabolism. Pantothenic acid is the direct precursor for CoA, which is a central cofactor in the TCA cycle (crucial for energy generation) and fatty acid biosynthesis [44,45]. This metabolic enhancement likely improves the adaptability of fermentation agents to the low-oxygen, acidic storage environment. In addition, the promoted biosynthesis of aromatic amino acids (phenylalanine, tyrosine, and tryptophan) serves two purposes: supporting protein synthesis and providing substrates for secondary metabolites such as indole compounds, which may facilitate microbial quorum sensing and synchronized population growth [46]. Meanwhile, the downregulation of arginine metabolism reduces the reliance on the arginine decarboxylation pathway and prevents excessive pH reduction during storage [47,48]. The coordinated suppression of purine and sulfur metabolism appears strategically important because it slows the nucleic acid synthesis rates, facilitates the transition from active proliferation to maintenance metabolism, and ultimately delays the cellular senescence [49]. These interconnected metabolic adjustments collectively helped maintain high levels of microbial communities of the L9 + HYY yogurt during refrigerated storage.

Integrated analysis indicated that *L. paracasei* L9 abundance was associated with increased levels of amino acid-related metabolites and changes in lipid-related metabolites, consistent with enrichment of glycerophospholipid metabolism and pantothenate/CoA biosynthesis. In contrast, *L. acidophilus* LAC abundance correlated with modulation of amino acid biosynthesis pathways, including arginine and branched-chain amino acid metabolism.

## 4. Conclusions

This study demonstrated that the incorporation of different *Lactobacillus* strains significantly affected the yogurt fermentation dynamics and storage performance via distinct metabolic strategies. Specifically, *L. paracasei* L9 exhibited superior fermentation capabilities: it rapidly increased the TA from 25.20 ± 7.01 to 36.56 ± 3.47 °T within 3 h and shortened the fermentation endpoint by 0.5 h through enhanced lactose usage via its high-affinity transport system and complete Leloir pathway. The metabolomic analysis revealed that *L. paracasei* L9 achieved high biomass accumulation (8.4 lg CFU/g) by upregulating the glycerophospholipid metabolism and pantothenate/CoA biosynthesis, whereas *L. acidophilus* LAC used alternative survival strategies by modulating the arginine biosynthesis and branched-chain amino acid metabolism to withstand acidic conditions. During 21 days of refrigerated storage, L9 + HYY maintained exceptional microbial stability (>9.0 lg CFU/g) through the coordinated upregulation of the aromatic amino acid biosynthesis and suppression of the purine/sulfur metabolism, but the viability of LAC significantly decreased to 6.02 lg CFU/g. These findings provide crucial insights into strain-specific metabolic adaptations and offer a scientific foundation to develop advanced probiotic yogurts with optimized fermentation efficiency and shelf-life stability.

## Data Availability

The data presented in this study are available within the article and its Supplementary Materials.

## Supplementary Materials

The following supporting information can be downloaded at https://www.mdpi.com/article/10.3390/foods14172935/s1: Figure S1: Composition of non-volatile metabolites identified in yogurts by untargeted metabolomics.
